# A temporal dependency feature in lower dimension for lung sound signal classification

**DOI:** 10.1038/s41598-022-11726-3

**Published:** 2022-05-12

**Authors:** Amy M. Kwon, Kyungtae Kang

**Affiliations:** 1 Artificial Intelligence Convergence Research Center, Hanyang University, Ansan, 15588 Korea; 2 Department of Applied Artificial Intelligence, College of Computing, Hanyang University, Ansan, 15588 Korea

**Keywords:** Outcomes research, Computational science

## Abstract

Respiratory sounds are expressed as nonlinear and nonstationary signals, whose unpredictability makes it difficult to extract significant features for classification. Static cepstral coefficients such as *Mel*-frequency cepstral coefficients (MFCCs), have been used for classification of lung sound signals. However, they are modeled in high-dimensional hyperspectral space, and also lose temporal dependency information. Therefore, we propose shifted $\delta$-cepstral coefficients in lower-subspace (SDC-L) as a novel feature for lung sound classification. It preserves temporal dependency information of multiple frames nearby same to original SDC, and improves feature extraction by reducing the hyperspectral dimension. We modified EMD algorithm by adding a stopping rule to objectively select a finite number of intrinsic mode functions (IMFs). The performances of SDC-L were evaluated with three machine learning techniques (support vector machine (SVM), k-nearest neighbor (*k*-NN) and random forest (RF)) and two deep learning algorithms (multilayer perceptron (MLP) and convolutional neural network (cNN)) and one hybrid deep learning algorithm combining cNN with long short term memory (LSTM) in terms of accuracy, precision, recall and F1-score. We found that the first 2 IMFs were enough to construct our feature. SVM, MLP and a hybrid deep learning algorithm (cNN plus LSTM) outperformed with SDC-L, and the other classifiers achieved equivalent results with all features. Our findings show that SDC-L is a promising feature for the classification of lung sound signals.

## Introduction

Lung sounds are characterized by airflow resistance when they are produced within the chest cavity during the respiration cycle consisting of inspiration and expiration phases^1^. Lung sounds are primarily categorized into vesicular and adventitious sounds. Vesicular sounds are ‘normal breathing sounds’ such as tracheal, bronchial and bronchovesicular sounds^2^, and they generally occur between frequencies of 100 Hz and 1000 Hz, with a sharp drop at about 100–200 Hz^3^. Adventitious sounds are additional sounds being superimposed onto vesicular sounds, which are generally formed when the airflow is interrupted by pulmonary deficiency in the tracheobronchial tree due to lung tissue changes or positions of secretion^4–6^. The adventitious sounds show different spectral contents to the vesicular sounds. In particular, adventitious sound signals are represented differently at specific frequency bands, intensities and time durations in different pathological conditions^7^. Respiratory disorders are generally associated with more than one lung sound, which is a relevant indicator of pathological conditions. Auscultation is a noninvasive technique for diagnosing diseases based on those characteristics of the sounds, and it has become an effective tool diagnosing respiratory disorders in a clinical setting. However, the signal quality and format of the acquired sounds are often incomparable among different types of sensors^8^. This has become an obstacle in research development among different laboratories. In addition, the auscultation process is highly subjective; hence, the diagnostic accuracy may vary depending on the physicians’ experience and skills in differentiating various sound patterns^9^.

Several studies have made efforts to resolve the inconsistencies in research and diagnostic results by objectively quantifying the characteristics of lung sounds. These efforts can be divided into two main directions. The first direction aims to digitize analog lung sounds; and to computerize the signal processing by considering sampling frequency, amplitude resolution, bandwidth of the signal, and calibration procedures for objective assessments^4,10,11^. In the same vein, the computerized respiratory sound analysis (CORSA) is a multinational effort of more than 20 researchers from seven European countries developing guidelines for the standard procedure for recording respiratory sounds^9^. Owing to these efforts, the digitization procedures and signal processing techniques of lung sounds have become standardized to a certain extent considering the different types of sensors. The second direction is focused on classifying pathological sound patterns based on quantified features, using modern techniques of artificial intelligence^6,12–16^. Typical machine learning techniques such as support vector machine (SVM) and random forest (RF) have been consistently used in most previous studies, and deep learning techniques such as artificial neural networks (ANN) and convolutional neural networks (cNN) have also recently been applied. However, there has been no significant improvement in selecting better features for classification even though the classification performance can be significantly influenced by them. Although computerized signal processing enables high resolution with reduced noise, it is still challenging to extract significant features from nonstationary signals such as lung sounds and to classify the sound signals as relating to diagnostic conditions.

A significant feature plays an essential role in classification, but there have been only limited types of features for classification of lung sound signals. In general, cepstral based features, such as *mel*-frequency cepstral coefficients (MFCCs) have been commonly used^2,14,17–19^. MFCCs are obtained by applying a discrete cosine transform to a number of coefficients from filter banks. Because the *mel*-filter is sensitive to small changes in lower frequencies, similar to the human hearing system, it is widely applied in speech recognition fields. Moreover, wavelet coefficients and short-time Fourier transformation have been used for lung sound classification in some studies^2,20^; however, for respiratory segments, MFCCs have been reported to be more effective in classifying abnormal breathing events, such as cough, in comparison with wavelet coefficients^2^. In addition, Fourier transformation is known to be less effective in extracting information from nonstationary signals such as lung sounds. Meanwhile, power spectrum^21^ and summary statistics^22,23^, such as kurtosis and quantiles, have been used to detect abnormal breathing sounds, with fair performance results. However, unlike in other topics, there is still no standard features that represent lung sounds with a limited variety. Thus, it is imperative to identify a new feature to characterize lung sounds better.

In this study, we suggest shifted-$\delta$ cepstral coefficients in lower-subspace (SDC-L) as a novel feature to characterize lung sounds. The shifted-$\delta$ cepstral coefficients (SDC) have been used to represent different levels of energy in vowels in speech recognition, and we applied SDC after reducing the hyperspectral dimension according to the empirical mode decomposition (EMD) algorithm to avoid deteriorating the classification performance due to high dimensionality. The remainder of this paper is organized as follows: we introduce the properties of a new feature, SDC-L, and of reference feature, MFCCs. Then, we describe the database used in the study, and highlight quantitative results in terms of precision, recall, accuracy and F1 score. Finally, we summarize our work by briefly discussing its strengths and limitations along with future research plans.

## Methods

Lung sounds are composed of complicated nonlinear and nonstationary multi-scale signals. Although these signals are denoised in advance, they are mostly unpredictable, which makes the extraction of significant features difficult. However, the features considerably affect performances of classifiers. Features are generally derived from the hyperspectral domain or a combination of temporal and hyperspectral domains rather than the temporal domain because linearity can be preserved in short time intervals, which are typically used to generate cepstral features in hyperspectral domain. However, most hyperspectral features lie in a high dimensional space and increased dimensionality can deteriorate classification performance^24^. As a solution, dimension reduction of cepstral features may improve the classification performance. demir successfully improved the classification accuracy by reducing the dimension of hyperspectral image data using the EMD algorithm. Based on this literature, our study proposes a novel temporal dependency feature, SDC-L using the EMD algorithm for the same purpose as the previous study.

### *Mel*-frequency cepstral coefficients

**Figure 1:**
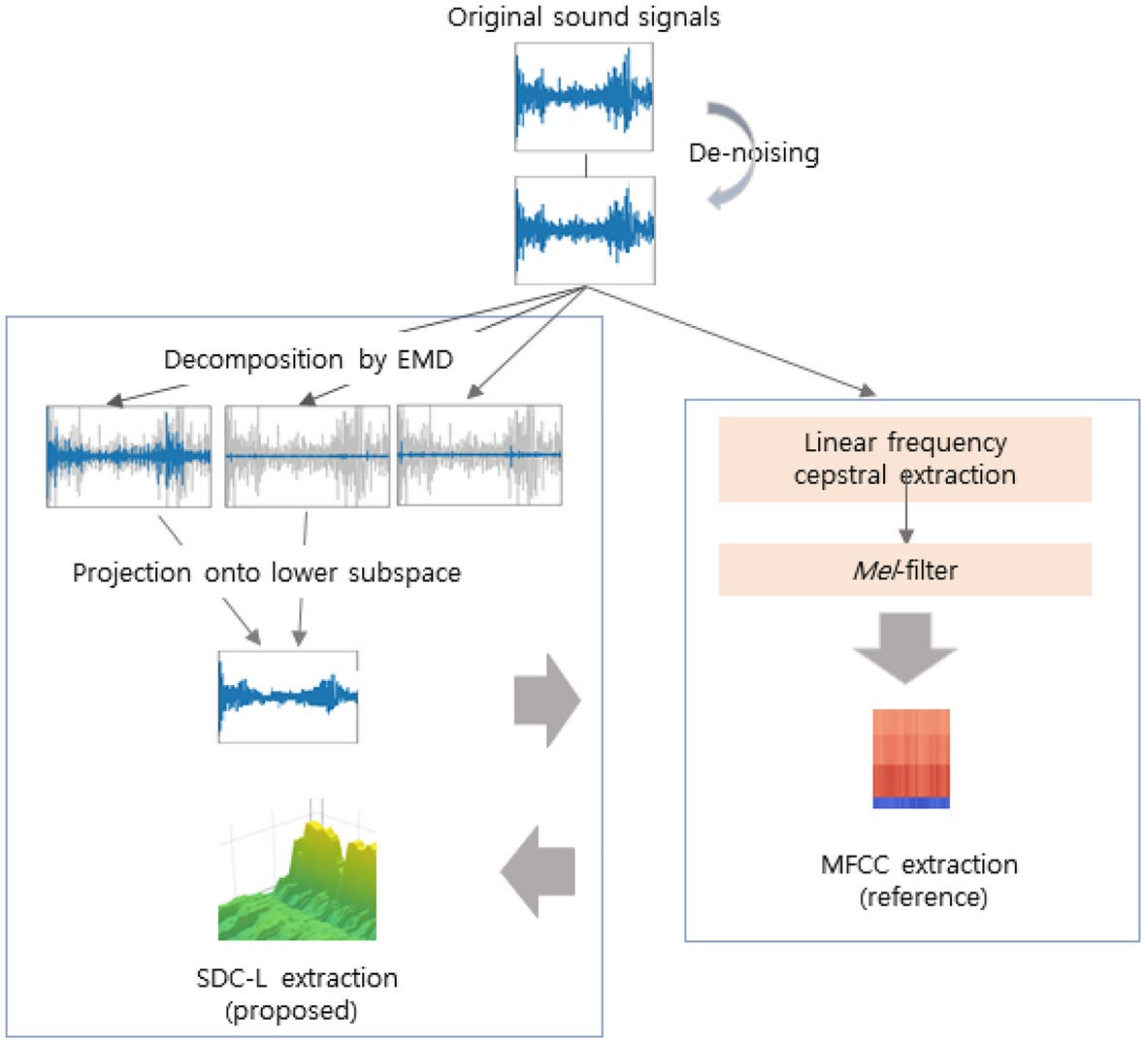
Block diagram of feature extraction.

As a filter-bank parameterization approach, MFCCs are widely used for classification to represent sound signals, and were also used as the reference feature in our experiments. MFCCs can be computed similarly to linear frequency cepstral coefficients but, they are imposed on the *mel*-scale frequency spectrum, which simulates the perceived frequency of sound signals in the human auditory system. The original frequency in the unit of Hz can be transformed to the *mel*-scale frequency using Eq. (1).

1 $$Mel(f) = 2595\times {\text{log}}\left( 1+\frac{f}{700}\right), $$

where ${\mathbf{f}}$ is the frequency. MFCCs are obtained by taking the cosine transformation to the logarithmic power of the *mel*-frequency. We extracted the first 13 parameters per sound signal using *Librosa* library in Python 3.9 The extraction procedure is illustrated in the right box in Fig. 1

### Shifted $\delta$-cepstral coefficients in lower-subspace

SDC-L is defined as a shifted $\delta$-cepstral feature extracted from a finite sum of intrinsic mode functions (IMFs). IMFs are products of EMD where EMD is an adaptive signal decomposition algorithm that sequentially divides a nonstationary and multi-scale signal, into IMFs and a residue until a constant and monotonic function with few extrema is obtained, which is no longer an IMF^25^. IMFs resemble filtered signals from the denoising process. The first IMF corresponds with a high-pass-filtered signal, and the other IMFs are similar to bandpass-filtered signals with the center frequency decreasing in an octave band manner like in a filter-bank approach^26^. Furthermore, IMFs form a basis for a subspace of dimensionality equal to the number of IMFs that are nearly orthogonal to each other^24^. Based on this characteristic, we refer to the shifted $\delta$-cepstral coefficients obtained from several IMFs as SDC-L in our study. As illustrated in the left panel on Fig. 1, SDC-L is generated by projecting the sound signals onto lower hyperspectral subspace consisting of a finite number of IMFs, and the generation procedure is summarized as follows.

1. Signal decomposition
  The sound signals are decomposed into IMFs and residue by a sifting procedure according to the EMD algorithm in Eq (2) where $y_i(t)$ and $r_K(t)$ indicate the ith IMF and the residue, respectively.
  2 $$x(t) = \sum _{k=1}^K{y(t)^{(k)}} + r_K(t) $$
  The EMD algorithm continues to search for the next IMF until no further IMF is found, and there is no schematic guideline for how to select a subset of IMFs. Our study newly modified the EMD algorithm by adding a stopping rule to it for this purpose (Table 1). Once the kth IMF is found, we non-parametrically test whether the hyperspectral surface is significantly different at the significance level of $\alpha$ to the former surface constructed by a discrete Fourier transform by means of the *L*2 distance according to the wild bootstrapping method^27^. The stopping rule is summarized in Table 2, and the test statistic can be calculated with Eq. 3.
  3 $${\mathscr{T}}_N = \sum _{l\le i < k \le L}~{\int {({\hat{m}}_i(x) -{\hat{m}}_k(x))^2\omega (x)dx}} $$
  where $\omega (\cdot )$ is a smooth and positive weight function and $m(\cdot )_i:{\mathscr{R}}^2 ~\rightarrow ~{\mathscr{R}}$ are unknown but smooth regression functions.

**Table 1:**
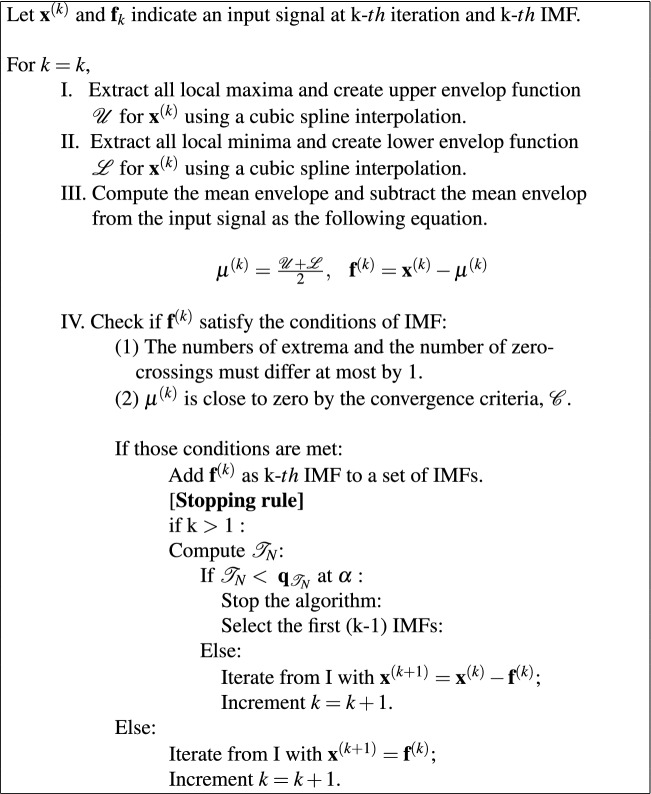
The modified EMD algorithm.

**Table 2:**
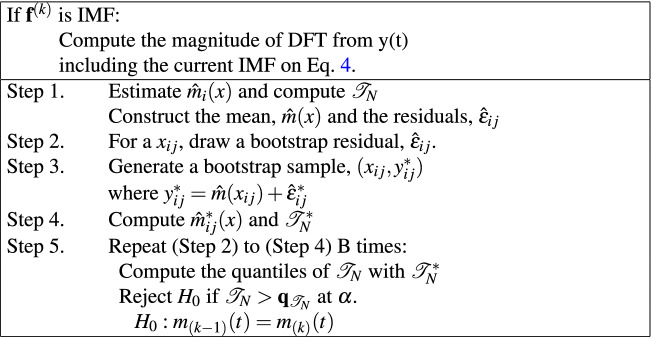
The stopping rule: determination of K.
2. Signal reconstruction
  The signals were reconstructed from the first IMF to the Kth IMF using Eq (4) according to the modified EMD algorithm with the stopping rule (Table 1). In the case of a discrete signal of length N, the number of IMFs is computed as $\log _2N$ at most: the dimensionality becomes much smaller than that of the original signal^24^. Thus, the reconstructed signals with the chosen K IMFs are naturally spanned in the low dimensional subspace with the IMFs forming an approximately orthonormal basis. The hyperspectral dimensional patterns based on discrete Fourier transform (DFT) by the ith IMF is shown in Experiment I in Section 3.
  4 $$y(t) = \sum _{i=1}^{k}{y_i(t)} $$
3. SDC extraction from low-subspace
  A SDC is a relatively new feature that has not been applied in the biomedical field yet. The main advantage of the SDC is its ability to incorporate additional temporal information, spanning multiple frames. $\delta$-cepstral coefficients have often been used as a static feature to add temporal dependency to a sequence of cepstral coefficients^28^. SDC is computed by linking these $\delta$-cepstral coefficients in multiple nearby frames, and its performance is superior to $\delta$-cepstral coefficients^29^. $\delta$-cepstral coefficients are expressed as Eq. (5) where ${\mathscr{C}}[t]$ indicates a sequence of cepstral coefficients, and t and d indicate the tth frame and lag size, respectively.
  5 $$\delta [t] = {\mathscr{C}}[t+d] - {\mathscr{C}}[t-d] $$
  SDC adds parameters of N, P and K to Eq. (5). N is the number of cepstral coefficients in each frame, K is the number of segments with concatenated $\delta$-coefficients, and P is the size of the time shift. A sequence of $\delta$-cepstral coefficients is computed from 0 to $K-1$ by shifting the time by the size of P for SDC in Eq. (6) where $j = 0,\ldots , (K-1)$.
  6 $$\delta [t+j\cdot P] = {\mathscr{C}}[t+j\cdot P+d] - {\mathscr{C}}[t+ j\cdot P-d] $$
  The scale of Eq. (6) is recently adjusted by $2\cdot d$; we used this adjusted SDC in this study. SDC-L is defined as an SDC feature in the lower subspace because a sequence of cepstral coefficients, ${\mathscr{C}}_r[t]$, comes from a reduced hyperspectral dimension that is formed by a finite sum of IMFs only. SDC-L is represented in Eq. (7) by replacing ${\mathscr{C}}(\cdot )$ with ${\mathscr{C}}_r(\cdot )$ in the original formula^30^, and we used MFCC as a sequence of static features in the study.
  7 $$\delta [t+j\cdot P] = \frac{\sum _{d=-d}^{d}{d\cdot {\mathscr{C}}_r[t+j\cdot P+d]}}{\sum _{d=-d}^{d}{d^2}} $$
  The original SDC is also extracted for performance comparison, and the patterns of SDC-L are shown by the diagnostic condition in Fig. 2.

**Figure 2:**
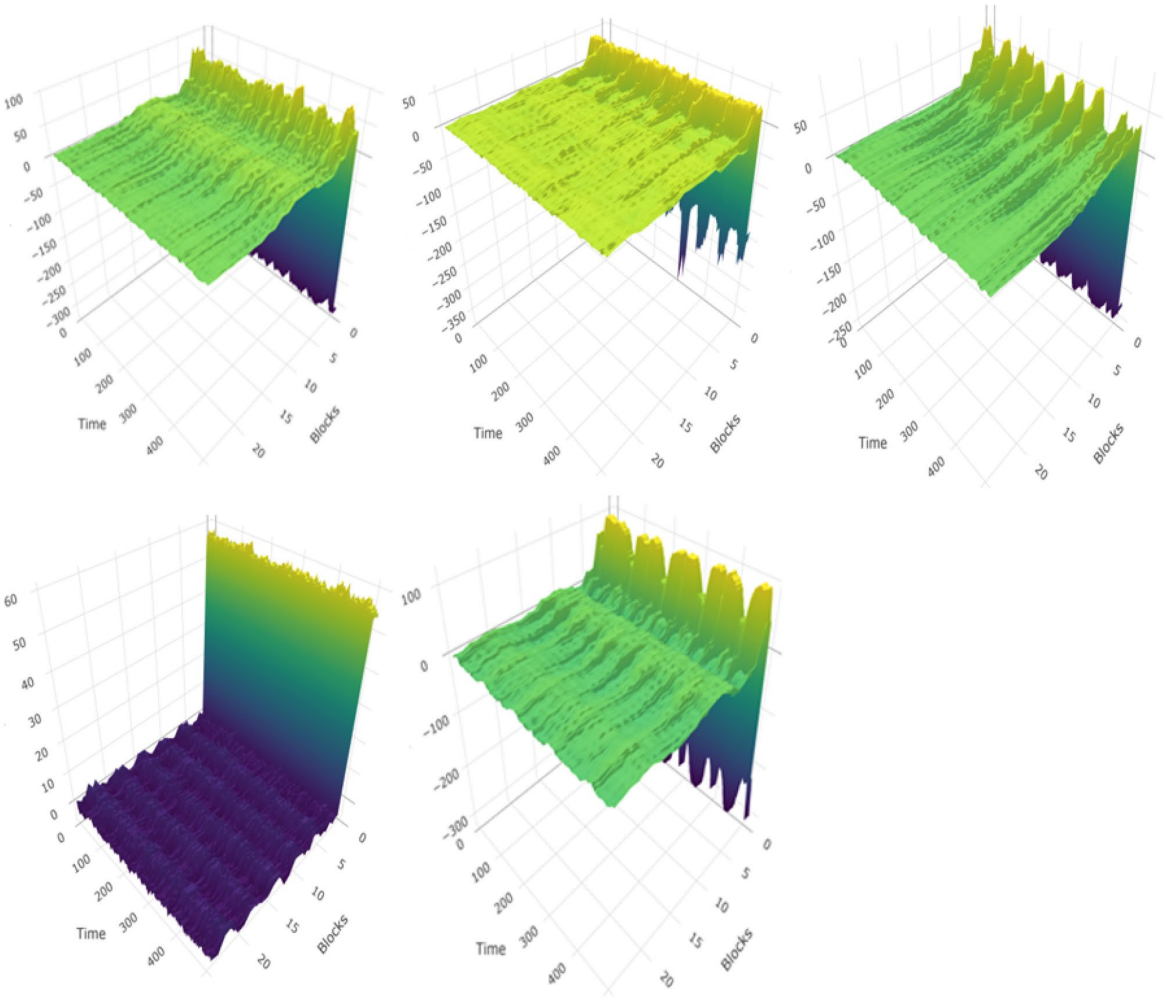
The patterns of SDC-L by the diagnostic condition : Healthy, URTI, COPD on the first row, and Pneumonia and Bronchiolitis on the second row.

### Classification algorithms

Numerous attempts have been made to classify lung sounds using machine learning techniques. According to a systematic review article that reported artificial intelligence techniques with lung sounds from 1982 to 2012^31^, a total of 39 studies used artificial intelligence techniques to classify the subjects based on the patterns of lung sounds. Moreover, after excluding the studies with sample sizes of less than 30 subjects from the listed studies, only 18 studies (approximately 46%) remained. Most studies classified the subjects as normal or with pathological conditions, and the pathological conditions were often replaced with abnormal breathing events such as wheeze and crackle^32,33^. For the analysis techniques, hidden markov model^34,35^, *k*-nearest neighbors (*k*-NN)^14,36^ and feedforward neural networks, wherein connections between nodes do not make a cycle, were used. The classification accuracies ranged from 69.59 to 98.34%^31^. Recently, SVM was used to classify the frequency bands of pulmonary sounds^37^, and RF was also utilized to detect wheezing sounds with 92.7% sensitivity^38^. Although the classification accuracy seems to be fair, these techniques may not be directly comparable owing to various features and different portions of the training data. To compare the performances between SDC-L and MFCCs, we first selected three machine learning methods, namely: SVM, *k*-NN and RF, which have been consistently applied to lung sounds. Additionally, we used two standard deep learning algorithms: multilayer perceptron (MLP) algorithm and cNN. In addition, we conducted a hybrid deep learning technique combining cNN with long short term memory (LSTM), which has most recently been applied to lung sound classification^39^.

## Results

### Data sources

We used the audio samples from the International Conference on Biomedical and Health Informatics respiratory sound database (2017) which was created by two research teams in Portugal and in Greece over a period of 7 years. The database contains respiratory sound recordings from clinical and non-clinical settings and is used to develop algorithms for sound classification^40^. A total of 6898 respiration cycles, which were acquired from 126 subjects and 920 sound recordings, were available in the dataset. Each cycle was annotated separately as a binary form of 0 and 1 for the two events of crackles and wheeze by trained clinical experts. Subjects were originally labeled with 6 different types of diagnosis in addition to ‘healthy‘ label: these were asthma, bronchiectasis, bronchiolitis, chronic obstructive pulmonary disease (COPD), lower respiratory tract infection (LRTI), upper respiratory tract infection (URTI) and Pneumonia. However, the numbers of subjects belonging to asthma, bronchiectasis and LRTI groups were less than 5, and these subjects were excluded from the study. Finally, we used 6302 respiration cycles with 836 sound recordings as the data set, and 5 types of diagnostic conditions were used as multiclass labels for classification: URTI, COPD, pneumonia, bronchiolitis and healthy.

### Data processing

Respiratory sounds are meaningful indicators of pathological conditions, but most sound samples are highly noisy for various reasons. The quality of sound signals of some sound samples in the dataset that were recorded in non-clinical environments was deteriorated. In addition, heart sound signals from heart murmurs and the vibration of blood vessels in the cardiac cycle widely influence the frequency ranges; thus, the convoluted signals are challenging to interpret. High pass filters, such as finite impulse response filters, are commonly adopted to separate heart sound signals from lung sound signals by removing the signals with lower frequency ranges with a threshold frequency between 50 and 150 Hz^41^; however, lung sounds may begin from 20 Hz depending on certain conditions^42^. For that reason, this study used wavelet transform based stationary-nonstationary filter based on Daubechies wavelet function, which is a denoising algorithm using wavelet coefficients^43^, and *PyWavelets* library in Python 3.9.

### Experiments and results

#### Experiment I

We modified the EMD algorithm by adding a stopping rule to resolve the problem that a cepstral based feature in a high-dimensional hyperspectral space deteriorates classification performance. The main purpose of Experiment I is to examine the modified version of the EMD algorithm. The EMD algorithm is completely data-dependent, and IMFs have different frequency contents according to the local properties of the data^25^. To examine the effect of sub-space spanning by a finite number of IMFs, we randomly selected 50 samples, and conducted a stopping rule with 1000 bootstrapping at $\alpha = 0.05$ if a new IMF is found. DFT was conducted by *SciPy* module in Python 3.9, and the results are summarized in Table 3. In Table 3, the first two column show the test statistic and corresponding p-value when the number of IMFs is *K*, the other four columns show the classification performance of the SVM classifier with SDC-L, which was extracted from the first K IMFs where *K* = 2, 3, 4.

**Table 3:** Experiment result: determination of K.

K	Test results		Performance			
	${\mathscr{T}}_N$	*p* value	Accuracy	Precision	Recall	F1 score
2	4.32e+08	0.0199^**^	0.81	0.93	0.81	0.86
3	1.03e+14	0.1393	0.81	0.93	0.81	0.85
4	3.93e+09	0.2139	0.81	0.93	0.81	0.86

According to Table 3, the mean surface of hyperspectral subspaces was significantly different between *K* = 1 and *K* = 2, but there were no further significant differences although the number of IMFs increased. In addition, we confirmed that the classification performances were not improved by increasing the number of IMFs. Therefore, we concluded that the subspace spanning by the first 2 IMFs is enough to define the hyperspectral dimension.

#### Experiment II

We extracted three different feature sets for Experiment II: MFCC, SDC and SDC-L. MFCC is a reference feature, and SDC is the shifted-$\delta$ cepstral coefficients in the original dimensional space. SDC-L is the proposed feature whose hyperspectral dimension is reduced by the first 2 IMFs as determined by Experiment I. The purpose of Experiment II is twofold: to compare the performance of two different types of features, MFCC and SDC, and to compare the performance between MFCC, a reference feature and SDC-L, the proposed feature in this study. All features were independently extracted from sound samples as illustrated in Fig. 1. As reference, the first 13 MFCCs were chosen with a hop size of 512 ms per signal, and they were also used as static features for both SDC and SDC-L. For SDC and SDC-L, both the block size and time shift parameters were set to 2. With these independent feature sets, we compared the performances of six different classifiers, three machine learning techniques, two standard deep learning algorithms and one hybrid deep learning technique, in terms of accuracy, precision, recall and F1 score as follows.

8 $${\text{Accuracy}}=  \frac{(TP+TN)}{(TP+TN+FP+FN)} $$

9 $${\text{Precision}}=  \frac{(TP)}{(TP+FP)} $$

10 $${\text{Recall}}=  \frac{(TP)}{(TP+FN)} $$

11 $${\text{F1  score}}=  \frac{2\cdot ({\text{Precision}}\cdot {\text{Recall}})}{({\text{Precision}}+{\text{Recall}})} $$

where TP and TN are the number of true positive and negative subjects, respectively, and FP and FN are the number of false positive and negative subjects, respectively.

**Figure 3:**
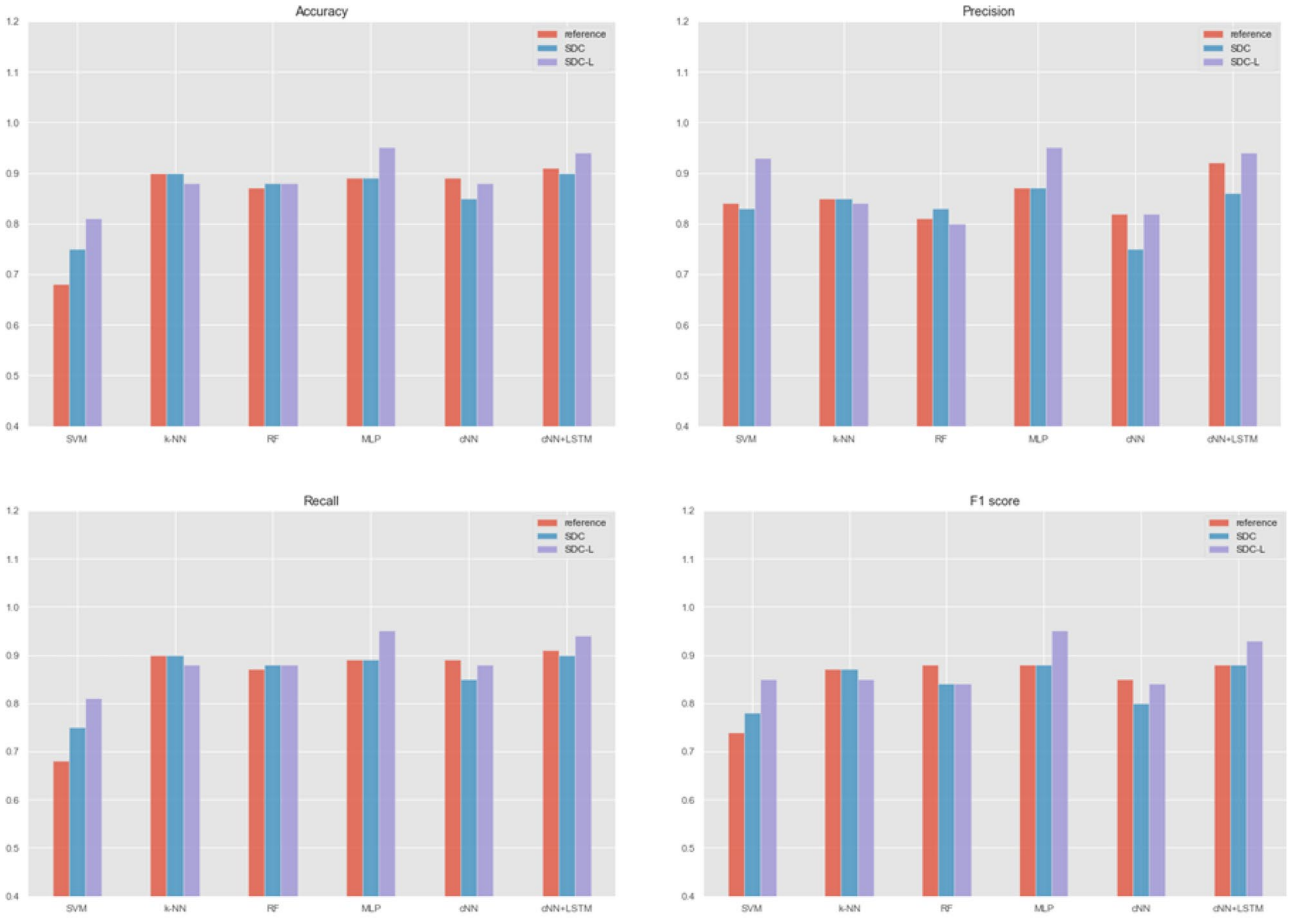
Performance comparison (MFCC vs. SDC vs. SDC-L).

**Figure 4:**
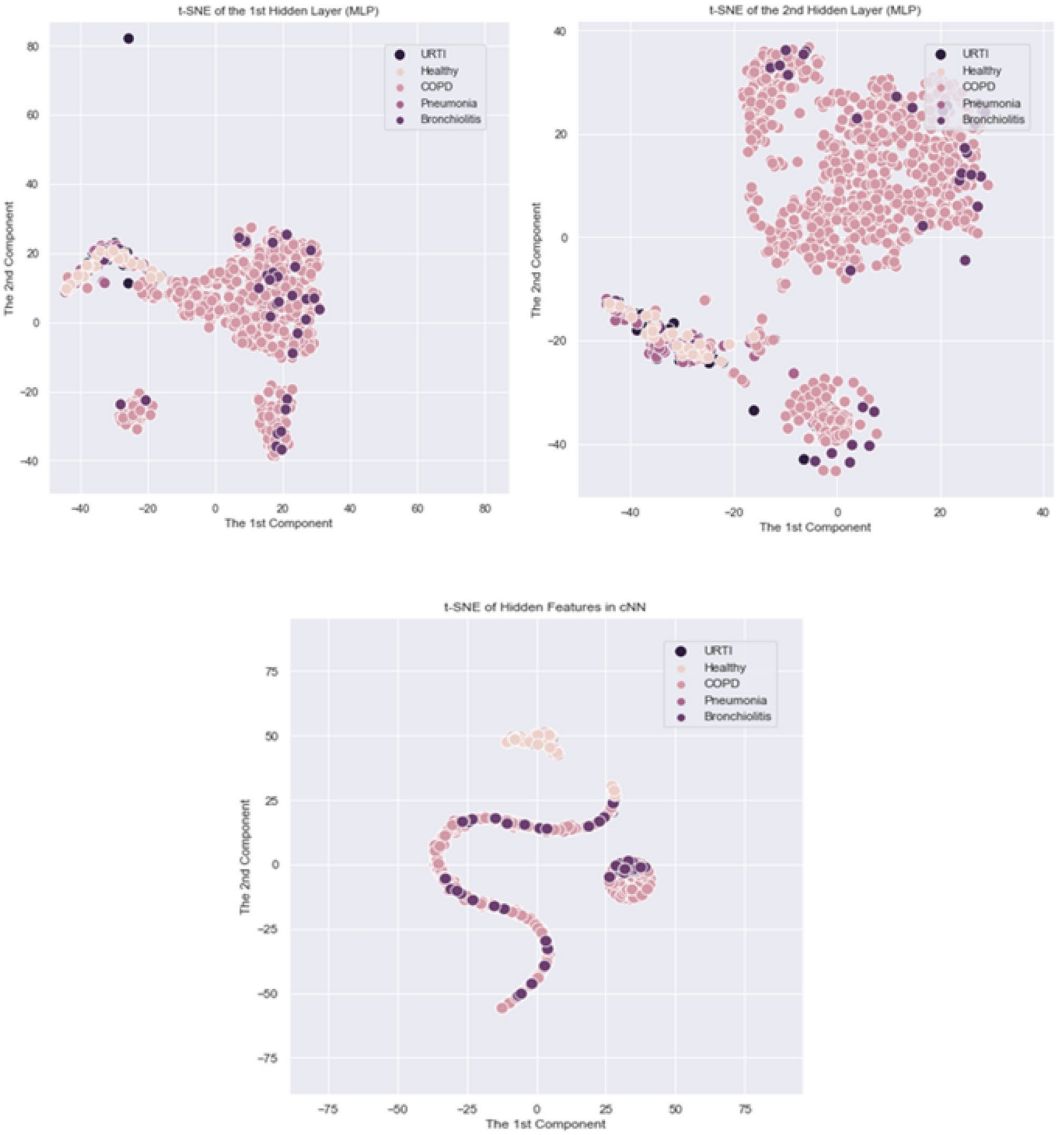
t-SNE patterns of Latent Features using SDC-L: The plots on the upper panel show t-SNE patterns of the latent features on the first hidden and the second hidden layers in MLP, respectively. The plot on the lower panel shows t-SNE pattern of the latent feature of cNN.

The performance results are summarized in Table 4. According to Table 4, all classifiers except cNN showed similar or better performances with the time dependency features of SDC and SDC-L in comparison with MFCC. In the case of cNN, the overall performances of SDC were slightly lower than MFCC, but the performances between SDC-L and MFCC were almost equivalent. In addition, all classifiers showed the best performance with SDC-L except *k*-NN. Particularly, SVM showed better performance with SDC-L than with SDC; and better performance with SDC than with MFCCs, a reference feature. Moreover, MLP showed the best performance with SDC-L obtaining 95% accuracy, precision, specificity and F1 score. We also conducted a hybrid deep learning algorithm by combining cNN with LSTM^39^. Its overall performances were improved in comparison with cNN alone regardless of the types of the features, but the tendency was preserved: It outperformed with SDC-L. The performance is compared graphically in Fig. 3. In addition, the implicit structures of the latent features in standard deep learning algorithms were visualized by t-SNE in Fig. 4, where t-SNE is a variation of stochastic neighbor embedding^44^, but it has been reported to show better visualization for high dimensional data^45^.

**Table 4:** Performance comparison.

Methods	Features	Accuracy	Precision	Recall	F1 score
SVM	Reference	0.68	0.84	0.68	0.74
	SDC	0.75	0.83	0.75	0.78
	SDC-L	0.81	0.93	0.81	0.85
*k*-NN	Reference	0.90	0.85	0.90	0.87
	SDC	0.90	0.85	0.90	0.87
	SDC-L	0.88	0.84	0.88	0.85
RF	Reference	0.87	0.81	0.87	0.88
	SDC	0.88	0.83	0.88	0.84
	SDC-L	0.88	0.80	0.88	0.84
MLP	Reference	0.89	0.87	0.89	0.88
	SDC	0.89	0.87	0.89	0.88
	SDC-L	0.95	0.95	0.95	0.95
cNN	Reference	0.89	0.82	0.89	0.85
	SDC	0.85	0.75	0.85	0.80
	SDC-L	0.88	0.82	0.88	0.84
cNN + LSTM	Reference	0.91	0.92	0.91	0.88
	SDC	0.90	0.86	0.90	0.88
	SDC-L	0.94	0.94	0.94	0.93

SVM, support vector machine; *k*-NN, *k*-nearest neighbors; RF, random forest; MLP, multi-layer perceptron; cNN, convolutionary neural network, LSTM, long short term memory.

#### Computational costs

We estimated the computational costs to extract the proposed feature according to the execution time on average. The machine idle time and hardware latency time were ignored for estimation. The task execution time (TET) was considered, and measured by *time* module in Python 3.9. We used CUDA vesion 11.4 computing platform with Nvidia GeForce RTX 2070 Graphic driver. The average TETs per 50 fragments with 882,000 bits per sample for EMD extraction, reconstruction and SDC-L computation were 7980.3076 s, 0.0808 s and 2.9929 s, respectively.

## Discussion

Respiratory sounds are meaningful indicators of pathological conditions, but the modern practice of auscultation in the real world has some limitations. Therefore, digitization of analog sounds and objective assessment based on machine learning techniques have consistently been performed. However, lung sound signals are characterized as nonstationary and non-periodic signals; this creates difficulties for extracting significant features for classification. Mostly, static features such as MFCCs, have been used. These features do not contain temporal dependency information and are generally high-dimensional, which may deteriorate the classification performance. Therefore, it is crucial to develop better features to improve the classification performance. For that reason, this study proposed a novel feature, SDC-L, which contains temporal dependency information of multiple nearby frames in a reduced hyperspectral dimension. According to our experiments, all classifiers showed better or equivalent performances with SDC-L except *k*-NN. In particular, SVM and MLP outperformed with SDC-L in comparison with the static feature of MFCC. In addition, we suggested a schematic procedure, adding a stopping rule to the EMD algorithm, to select a finite number of IMFs under the inference framework to reduce the original dimension to a subspace. The stopping rule worked successfully and we demonstrated that the first 2 IMFs are enough to explain the feature by this stopping rule in the experiment. The result is consistent with the previous study concluding that all IMFs except the first IMF are similar to band-pass filtered signals^26^. However, SDC-L also has some limitations. The number of features per frame could increase in proportion to the size of the block; this requires careful treatment if the maximum number of features is limited in the classification method or if a signal fails to find IMFs. Nevertheless, classifiers using SDC-L showed promising performance. As features play a significant role in classification, SDC-L is worthy of further study as a feature to identify distinctive characteristics of lung sound signals for diagnostic purposes. In a future project, we will explore the effect of temporal dependency on the classification performance and plan to derive a compact feature from SDC-L.

## Data Availability

The datasets analyzed during the current study are available in the International Conference on Biomedical and Health Informatics respiratory sound database (2017) https://bhichallenge.med.auth.gr/.
